# Correction to: Hepatocyte growth factor inhibits TGF-β1-induced myofibroblast differentiation in tendon fibroblasts: role of AMPK signaling pathway

**DOI:** 10.1186/s12576-020-00778-7

**Published:** 2020-10-26

**Authors:** Qingbo Cui, Songbin Fu, Zhaozhu Li

**Affiliations:** 1 Pediatric Orthopedics Unit, Second Affiliated Hospital of Harbin Medical University, 246 Xuefu Road, Harbin, 150081 China; 2 Laboratory of Medical Genetics, Harbin Medical University, Harbin, China

## Correction to: J Physiol Sci (2013) 63:163–170 10.1007/s12576-013-0251-1

Following publication of the original article [1], the authors identified an error in Figs. 2a, 3a and 5a. These panels contain incorrect representative images of cell morphology because some small cell morphology images were placed in the wrong position during figure preparation. The experiments have been re-performed, and the correct versions of Figs. 2a, 3a and 5a are provided below.

**Fig. 2:**
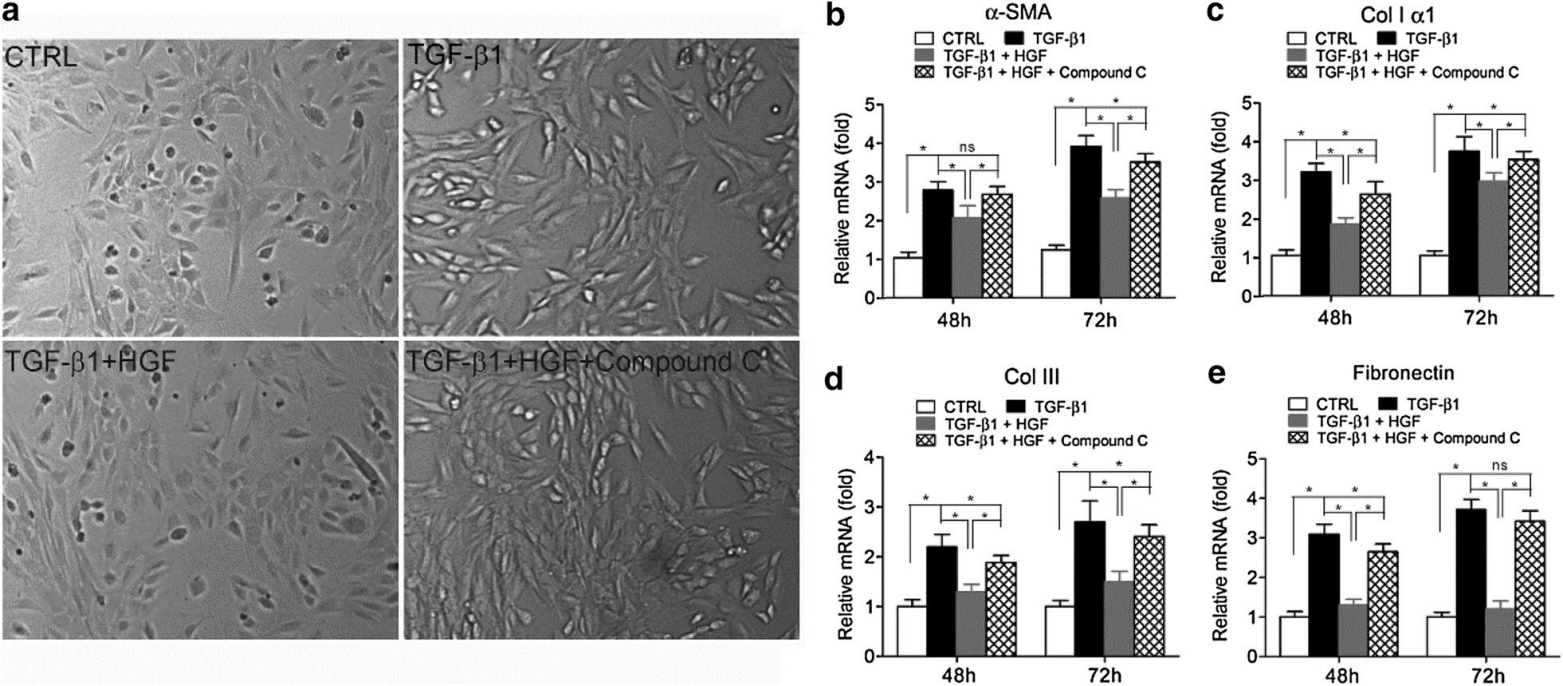
AMPK inhibitor compound C attenuated the inhibitory effect of HGF on the TGF-β1-induced myofibroblastic differentiation in tendon fibroblasts. **a** Typical cell morphology of tendon fibroblasts under stimulation by TGF-β1 and HGF. **b**–**e** Tendon fibroblasts were treated with TGF-β1 (10 ng/ml), TGF-β1 (10 ng/ml) + HGF (20 ng/ml), or TGF-β1 (10 ng/ml) + HGF (20 ng/ml) + compound C (20 μM) for 48 and 72 h. Then, the mRNA levels of α-SMA (**b**), Col I α1 (**c**), Col III (**d**), and fibronectin (**e**), four markers of myofibroblastic differentiation, were measure by real-time quantitative PCR analysis; β-actin was used as a housekeeping gene for reference. All data were normalized to β-actin expression (2^−∆∆Ct^ methods). *N* = 8. *P < 0.05

**Fig. 3:**
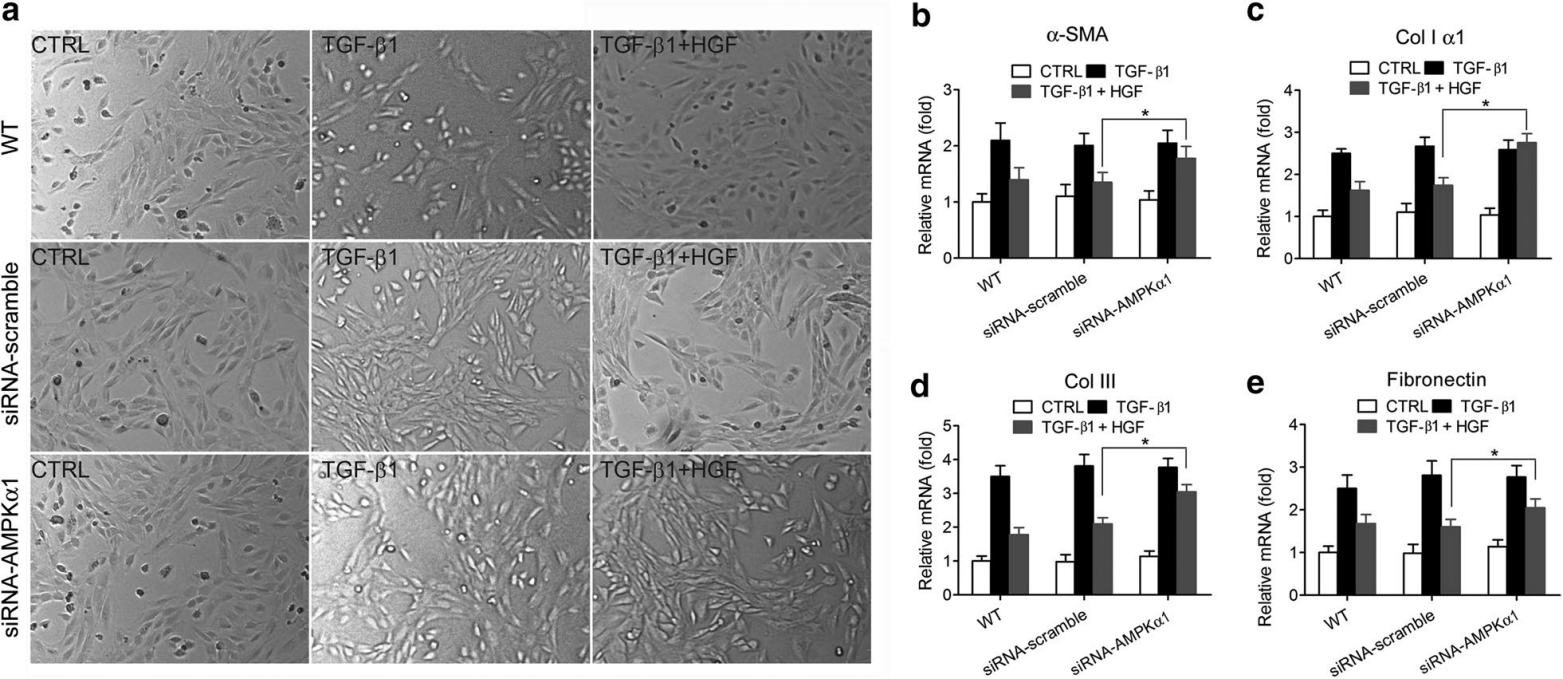
Knocking down of AMPKα1 disrupted the inhibitory effect of HGF on the TGF-β1-induced myofibroblastic differentiation in tendon fibroblasts. Wild-type tendon fibroblasts, scramble-siRNA-transfected tendon fibroblasts, and AMPKα1-targeting siRNA-transfected tendon fibroblasts were treated with TGF-β1 (10 ng/ml) or TGF-β1 (10 ng/ml) + HGF (20 ng/ml) for 72 h. **a** Typical cell morphology of control, siRNA-scramble transfected, and siRNA-AMPKα1 transfected tendon fibroblasts under stimulation by TGF-β1 and HGF. **b**–**e** The mRNA levels of α-SMA (**b**), Col I α1 (**c**), Col III (**d**), and fibronectin (**e**), four markers of myofibroblastic differentiation, were measure by real-time quantitative PCR analysis; β-actin was used as a housekeeping gene for reference. All data were normalized to β-actin expression (2^−∆∆Ct^ methods). *N* = 8. *P < 0.05

**Fig. 5:**
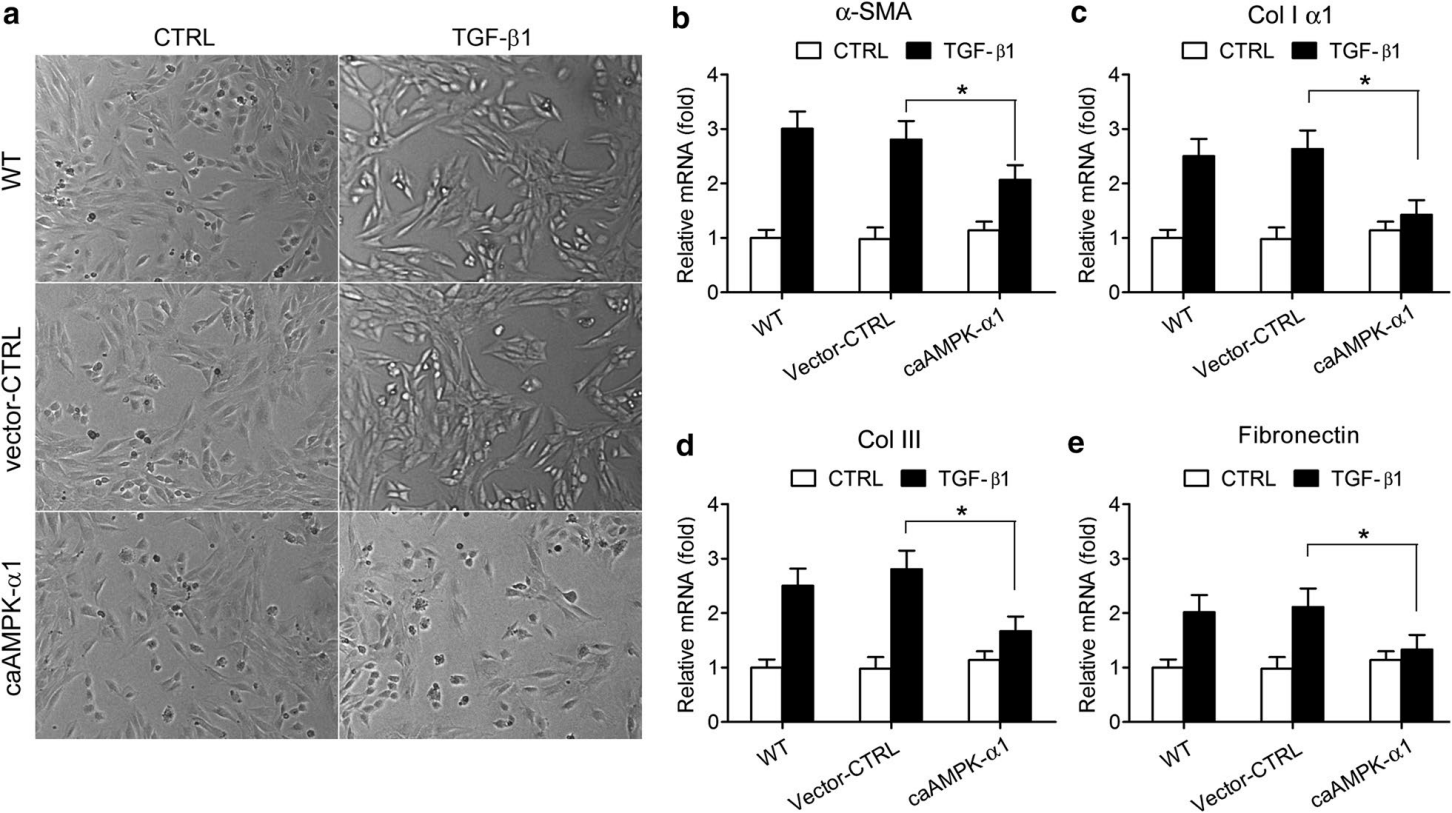
Overexpression of constitutively active AMPKα1 plasmid (caAMPKα1) mimics the inhibitory effect of HGF on the TGF-β1-induced myofibroblastic differentiation in tendon fibroblasts. **a** Typical cell morphology of control, vector transfected, and ca-AMPKa1 transfected tendon fibroblasts under stimulation by TGF-β1. **b**–**e** The mRNA levels of α-SMA (**b**), Col I α1 (**c**), Col III (**d**), and fibronectin (**e**) were measure by real-time quantitative PCR analysis; β-actin was used as a housekeeping gene for reference. All data were normalized to β-actin expression (2^−∆∆Ct^ methods). *N* = 8. *P < 0.05
